# Hardness Distribution and Growth Behavior of Micro-Arc Oxide Ceramic Film with Positive and Negative Pulse Coordination

**DOI:** 10.3390/nano14100842

**Published:** 2024-05-10

**Authors:** Haomin Li, Shiqin Kong, Zhiming Liu, Zhenxing Wang, Yingsan Geng

**Affiliations:** State Key Laboratory of Electrical Insulation and Power Equipment, Xi’an Jiaotong University, Xi’an 710049, China

**Keywords:** micro-arc oxidation, growth behavior, bipolar pulse, hardness distribution, synergistic effect

## Abstract

Micro-arc oxidation (MAO) is a promising technology for enhancing the wear resistance of engine cylinders by growing a high hardness alumina ceramic film on the surface of light aluminum engine cylinders. However, the positive and negative pulse coordination, voltage characteristic signal, hardness distribution characteristics of the ceramic film, and their internal mechanism during the growth process are still unclear. This paper investigates the synergistic effect mechanism of cathodic and anodic current on the growth behaviour of alumina, dynamic voltage signal, and hardness distribution of micro-arc oxidation film. Ceramic film samples were fabricated under various conditions, including current densities of 10, 12, 14, and 16 A/dm^2^, and current density ratios of cathode and anode of 1.1, 1.2, and 1.3, respectively. Based on the observed characteristics of the process voltage curve and the spark signal changes, the growth of the ceramic film can be divided into five stages. The influence of positive and negative current density parameters on the segmented growth process of the ceramic film is mainly reflected in the transition time, voltage variation rate, and the voltage value of different growth stages. Enhancing the cathode pulse effect or increasing the current density level can effectively shorten the transition time and accelerate the voltage drop rate. The microhardness of the ceramic film cross-section presents a discontinuous soft-hard-soft regional distribution. Multiple thermal cycles lead to a gradient differentiation of the Al_2_O_3_ crystal phase transition ratio along the thickness direction of the layer. The layer grown on the outer surface of the initial substrate exhibits the highest hardness, with a small gradient change in hardness, forming a high hardness zone approximately 20–30 μm wide. This high hardness zone extends to both sides, with hardness decreasing rapidly.

## 1. Introduction

The Piston engine is the core power equipment of small aircraft. There has been a shift towards using lightweight aluminium alloy cylinder blocks instead of the traditional cast iron ones due to the requirements for lighter weight, longer lifespan, and higher efficiency. However, the hardness and wear resistance of aluminum alloys are relatively insufficient. Even with strengthening, it is challenging to achieve mechanical properties and wear resistance comparable to those of cast iron materials [1]. Therefore, the key to the development and application of lightweight aluminum alloy engine technology lies in the strengthening and modification of the surface of the aluminum alloy cylinder block or liner. A promising approach involves the in-situ growth of an alumina ceramic film on the surface of the aluminium alloy through micro-arc oxidation. This method offers several advantages over the current practice of depositing a Ni-SiC coating on the surface of the aluminum alloy cylinder. The alumina ceramic film not only significantly enhances the surface hardness and wear resistance of the aluminum alloy, but the in-situ growth method also facilitates metallurgical bonding at the interface with the aluminum alloy substrate through atomic mutual diffusion. This effectively addresses the issue of the relatively weak bonding force associated with coating deposition [2,3]. Moreover, the irregular random microporous structure on the surface of the alumina provides superior oil storage and lubrication performance compared to the regular reticular structure on the surface of the cast iron cylinder liner. Therefore, it is of great significance to construct a wear-resistant coating with high hardness and certain thickness on the surface of the aluminum alloy in situ using the micro-arc oxidation method. This will undoubtedly enhance the performance of lightweight aluminum alloy engines.

The wear resistance of ceramic films produced by micro-arc oxidation is intrinsically linked to the hardness of the layer [4]. The quality and performance of the micro-arc oxidation film are primarily influenced by factors such as the electrolyte composition, substrate, and electrical parameters. Extensive researches have been studied both domestically and internationally on the impact of micro-arc oxidation process parameters on the hardness of ceramic films. The ceramic film formation mechanism of the micro-arc oxidation film on the surface of the aluminum alloy varies across different electrolyte systems, and the content of alloy elements in the matrix can affect the composition and phase structure of the film. Consequently, Hardness is also different [5]. Xin T. et al. conducted a study where they added sodium silicate and sodium aluminate to a potassium hydroxide solution, respectively [6]. The results demonstrated that the film formed in the silicate system was thicker and had a larger roughness, while the ceramic film formed in the sodium aluminate system exhibited higher hardness and a greater dense layer ratio. Furthermore, the concentrations of sodium silicate and sodium aluminate were found to significantly influence the thickness, hardness, and dense layer ratio of the ceramic film. In a separate study examining the effects of different substrate materials on the properties of micro-arc oxidation ceramic films, He Z. et al. discovered that the hardness of the micro-arc oxidation ceramic film was the lowest when a pure Al substrate was used [7]. In contrast, the hardness of the ceramic film corresponding to LY12 and LC4 was relatively high. Once the electrolyte and substrate compositions are established, the micro-arc oxidation power supply’s parameter settings become the primary determinants of the film’s performance. The specific electrical parameters include the power supply mode (constant current or constant voltage), voltage or current density, and the duty cycle and frequency of the pulse power supply mode. These parameters dictate the form of the applied current/voltage that flows across the metal workpiece’s surface. This influences the rate of film growth and the reaction effect of film formation, which in turn affects the hardness performance by modifying the final film’s phase composition and morphology. It is widely accepted that positive current/voltage primarily drives the growth of the oxide film, making positive output a common operating mode in micro-arc oxidation treatment. Recent studies have shown that the appropriate introduction of negative current/voltage can adjust the film’s state, thereby influencing the its hardness. Hussein et al. conducted a study on the impact of negative current on the formation process of oxidation films in a silicate system [8]. The findings revealed that the introduction of negative current effectively suppressed large local spark discharges on the oxide film’s surface, enhancing the film’s compactness and overall quality. However, the film produced under bipolar current conditions was found to be relatively thin and brittle. Furthermore, when the ratio of negative to positive current *R* is greater than or equal to 1, a phenomenon known as ‘soft spark’ occurs. This involves a soft spark transition during micro-arc discharge, accompanied by a simultaneous voltage drop. Tsai et al. employed this method to inhibit the discharge channels and defects that form during the growth of ceramic membranes [9]. Their results indicated that compared to the non-soft spark discharge mode, the film formed in the soft spark discharge mode was thicker, the ratio of the dense layer to the porous layer increased, the number of defects in the film decreased, and the hardness of the film increased. This research provides valuable insights into the role of electrical parameters in micro-arc oxidation treatment and their impact on film properties.

To improve the microhardness of the micro-arc oxidation coating on aluminium alloy and refine the technology, researchers are working on designing the more effective micro-arc oxidation process. This approach is primarily focused on increasing the proportion of the hard phase in the micro-arc oxidation coating. It is determined that when the proportion of α-Al_2_O_3_ in the ceramic film reaches 70%, the microhardness of the ceramic film can be increased to 1800–2000 HV [10]. In contrast, the hardness of the micro-arc oxidation ceramic film containing 60% γ-Al_2_O_3_ is only 1400–1600 HV. Arrabal et al. effectively increased the content of α-Al_2_O_3_ phase in the outer layer of the ceramic film by incorporating α-Al_2_O_3_ particles, and improved the porosity and hardness of the ceramic layer [11]. J.-H Wang et al. conducted a study on the effect of the positive and negative current ratio on the structural characteristics of micro-arc oxidation ceramic coatings [12]. Their results indicate that as the positive and negative current ratio decreases, the phase content ratio and hardness of α-Al_2_O_3_ and γ-Al_2_O_3_ in ceramic coatings increase. Unfortunately, at present, the influencing factors of the relative proportion of α-Al_2_O_3_ and γ-Al_2_O_3_ in the micro-arc oxidation ceramic film are not systematic and comprehensive enough, which need to be further explored and studied.

The above analysis shows that improving the cathode pulse in both positive and negative pulses enhance the microstructure and hardness of the ceramic film. However, the internal influence mechanism of the alumina growth process and the hardness characteristics of the ceramic film under the positive and negative pulses are still unclear. This study aims to adjust the external positive and negative pulse intensity to investigate further. This adjustment enables the observation of the anode voltage change during the growth process. The key factors influencing the ceramic film’s micro structure are clarified, and the impact of different positive and negative pulse intensities on the hardness distribution characteristics of the ceramic film is established. Ultimately, the internal mechanism of the synergistic effect of positive and negative pulses on the growth and hardness distribution characteristics of the ceramic film is revealed. 

## 2. Materials and Methods

**Figure 1:**
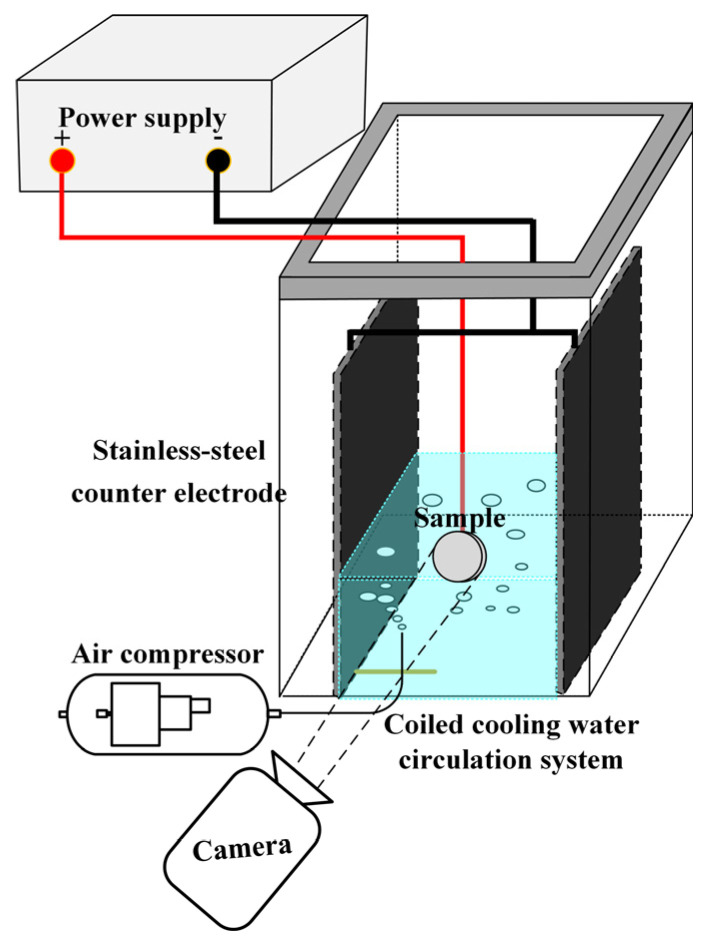
Schematic diagram of the experiment.

This study focuses on the preparation of a ceramic membrane on a 2024 aluminum alloy, which is commonly used as a material for cylinder liners in aero engines. Disc sample of aluminum alloy, with a size of ∅40mm × 8mm, was used for the experiment. The ceramic film was prepared through a process of micro-arc oxidation. This process was conducted in a plating tank, equipped with a compressed air stirring and water-cooling system. The tank features a transparent glass front, allowing for the observation of the sample surface’s discharge state. Inside the plating bath, two rectangular stainless steel electrodes were arranged in parallel. The sample was positioned centrally between these electrodes. A self-developed micro-arc oxidation power supply provided positive and negative pulses. The chemical composition of the sample (wt. %) is: (Cu 3.91, Mg 1.44, Mn 0.43, Fe 0.28, Si 0.14, Zn 0.05, Ti 0.03, Cr 0.01, and Al). Throughout the experiment, a camera was used to observe the surface discharge of the sample during the growth of the alumina. An oscilloscope recorded the voltage and current signals during the ceramic membrane’s growth. The experimental apparatus is shown in Figure 1.

During the experiment, the electrolyte was composed of 5 g/L liquid sodium silicate and 5 g/L potassium hydroxide, and the temperature during the preparation of the ceramic film was controlled below 30 °C. The duration of micro-arc oxidation is 100 min. A constant current control method was employed in this study to yield a ceramic film with high hardness. This was achieved by introducing a strong cathode pulse induced soft spark discharge. The output waveforms of both positive and negative pulses, had a duration of 1.6 ms each, with an intermission time of 0.4 ms between them. The anode current density (*J*_a_) was set at four levels: 10, 12, 14, and 16 A/dm². Additionally, the ratio R of the cathode current density (*J*_c_) to the anode current density (*J*_a_) was divided into three grades: 1.1, 1.2, and 1.3. The sample number and the positive and negative pulse current densities were set as per the details provided in Table 1.

Before the experiment, the aluminum alloy sample was ultrasonically cleaned to remove impurities such as oil stains on its surface, and then the sample was washed with alcohol and dried. After the experiment, the thickness of the micro-arc oxidation ceramic film was measured using a PHYNIX Surfix SX eddy current thickness gauge. To ensure data accuracy, each position was measured a minimum of five times, with the average value being recorded. The original surface of the aluminum substrate served as the marker line for determining the internal and external growth of the ceramic film. The surface and cross-section morphology of the film were examined using a SU3500 tungsten filament scanning electron microscope operating at 15 kV. The phase composition, crystallinity, and grain size of the micro-arc oxidation film were analyzed using a D8 AD-VANCEA25 X-ray diffractometer (CuKα, 40 mA, 40 kV, with a 2θ scanning range of 20° to 90°). The surface roughness of the film was measured using a Leeb432 surface roughness meter. Eight points on the ceramic film’s surface were measured, and the average value was calculated to determine the final roughness. The micro-Vickers hardness distribution of the coating section was obtained using a TMVS-1 microhardness tester (load 1.96 N, holding pressure 15 s). This methodology ensures a comprehensive and accurate analysis of the properties of the ceramic film.

## 3. Results

### 3.1. Micro-Plasma Discharge Behavior and Ceramic Film Voltage Characteristics

**Figure 2:**
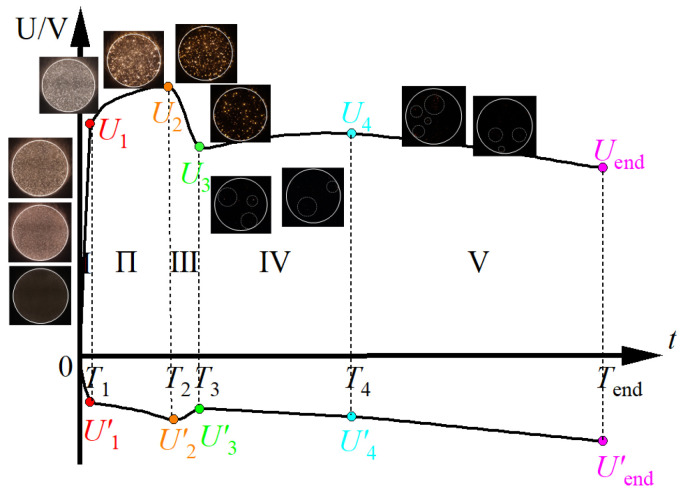
Positive and negative voltage amplitude curve and micro-plasma discharge phenomenon during process. *T*: transition time, *U*(*U*′): The voltage value corresponding to the transition time.

The micro-plasma discharge occurring on the surface of aluminum alloy constitutes a complex dielectric barrier discharge, which serves as the defining characteristic of micro- arc oxidation (MAO). This discharge phenomenon includes breakdown of the alumina film in both solid and gas mediums due to redox reactions. Multiphase surface discharges within micropores may also occur, which can directly influence mass transfer and phase change dynamics during the ceramic film growth process. Consequently, the growth state of the ceramic film can be effectively elucidated by monitoring plasma discharge behavior and dynamic voltage variations applied to the sample [13]. The micro-plasma discharge behavior and the dynamic voltage curve under the action of positive and negative pulses during the growth of the ceramic film of sample # 9 are shown in Figure 2. By examining the evolution of the positive voltage signal and the characteristics of micro-plasma discharge, the growth process of the ceramic film can be delineated into five distinct stages. 

During the initial stage (Stage I), the positive voltage exhibits a rapid escalation, reaching 430V within a minute, marking the conclusion of the initial rapid rise phase. Simultaneously, the negative voltage undergoes a rapid increase to approximately -80V in a nearly linear fashion. Notably, during this period, swiftly moving micro-plasma discharge spots emerge on the sample’s surface, albeit with relatively weak discharge sparks. The duration of most discharge spots does not exceed ten microseconds. This phase indicates the surface passivation process and the initial formation of the alumina film [14,15].

Stage II spans from 1 min to 21 min, during which the rate of voltage rise for both positive and negative electrodes diminish to less than one-fifth of that observed in the preceding stage. This period is characterized by a gradual and slow voltage rise. Notably, large-scale discharge spots begin to manifest on the sample’s surface, accompanied by an increase in the duration of discharge spots. Within this stage, discharge predominantly occurs in weak areas of insulation within the ceramic layer, resulting in two distinct discharge characteristics. The initial symptom is the presence of large orange discharge spots that tend to reoccur in the same areas and gradually diminish over time. The second characteristic comprises small, short-lived white bright discharge spots, exhibiting random breakdown positions and significant jumps. The collective discharge spot phenomenon demonstrates the coexistence of short-term, freely moving small spots and long-term, slowly moving large spots on the sample surface. The thickening mechanism of the ceramic film in this stage primarily involves deposition sintering, with amorphous alumina undergoing rapid crystallization under discharge conditions. Previous research indicates that γ-alumina remains the dominant phase within the ceramic film.

21 min later, a notable decrease in positive voltage ensues, stabilizing at 23 min with an approximate reduction of 20%. This sequence delineates stage III, characterized by a sudden drop in both positive and negative voltages. Stage III typically denotes the transition of micro-arc discharge into the ‘soft spark’ discharge mode [16]. Within this stage, plasma discharge spots diminish significantly, accompanied by the rapid disappearance of short-term, freely moving small spot discharges, and a gradual decrease in the number of long-term, slowly moving large spot discharges until their cessation.

**Figure 3:**
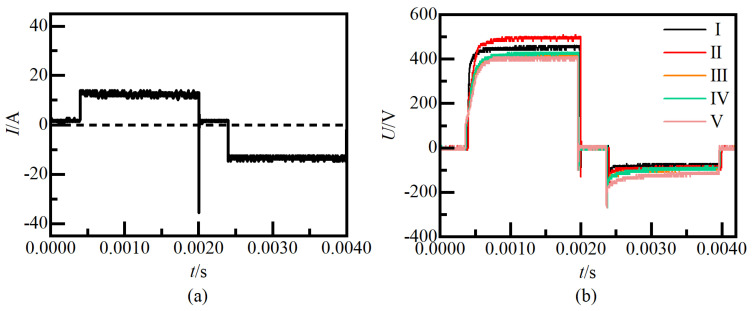
Waveforms of voltage pulses at the output of the power supply at different processing times. (**a**) current waveform, (**b**) voltage waveform.

Subsequently, from 23 min to 55 min, stage IV unfolds, during which the positive electrode voltage undergoes slight directional changes, experiencing an increase of approximately 20 V. This stage is succeeded by stage V, spanning from 55 min until the conclusion of micro-arc oxidation, characterized by the gradual and sustained decrease in positive electrode voltage. During both stage IV and stage V, a gradual upward trend in negative voltage is evident, indicative of the ‘soft spark’ mode discharge characteristic. The stages are identified by sporadic weak micro-plasma discharges on the sample’s surface. These discharges appear as small spots with short durations, as shown in Figure 3 where the discharge positions are highlighted by circles. Notably, at this juncture, light emanates from within the sample, suggesting a transfer of micro-plasma discharge positions to the interior of the ceramic film, thereby resulting in the diminishing occurrence of penetrating discharge. Throughout these stages, the thickening mechanism of alumina ceramics undergoes a notable transition from deposition sintering to a combined process involving deposition sintering at the surface of the ceramic membrane and oxygen permeation at the base of the ceramic membrane. At the same time, the internal discharges within the ceramic film accelerate the crystallization and phase transformation of alumina.

Figure 3 shows the current and voltage pulse waveform recorded on sample #9 at the midpoint of each of the five stages during the ceramic film growth process. In constant current mode, the current does not change and the voltage changes passively. The Figure 3a illustrates the presence of burrs and spikes in the actual output current waveform. Notably, as the oxidation time increases, it is observed in the Figure 3b that the duration of the rising edge of the positive pulse elongates, while the falling edge of the negative pulse similarly extends. If one were to conceptualize the metal matrix, ceramic membrane, electrolyte, and stainless steel electrode system as an equivalent composite dielectric capacitor model within the circuit framework, the time constant of this equivalent circuit system progressively increases. Furthermore, considering the perspective of crystallization phase transition, the relaxation time of the alternating polarization process of the ceramic film also experiences an increase. Significantly, from stage I to stage V, subsequent to the decline in the positive voltage line, although the voltage may experience a rise, both the maximum rise value and the termination voltage are markedly lower than the voltage preceding the voltage drop. Conversely, the negative voltage exhibits a slow rise following the voltage drop, with the termination voltage surpassing the voltage level prior to the drop. 

### 3.2. The Voltage Variation Law of Ceramic Membrane Growth Process under Different Positive and Negative Pulses

**Figure 4:**
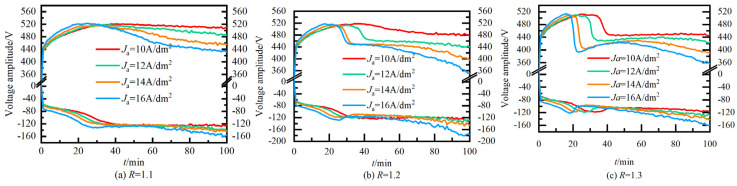
Voltage curve with different positive and negative pulse combination. (**a**) *R* = 1.1 (**b**) *R* = 1.2 (**c**) *R* = 1.3.

Figure 4 illustrates the voltage change curves of 12 sample groups during the preparation process under various combinations of positive and negative pulses. It is apparent from the diagram that the voltage curves exhibit significant differences across different values of *J*_a_ and R, indicative of distinct growth processes of alumina. Figure 4 shows the voltage change curves of 12 sample groups during the preparation process under various combinations of positive and negative pulses. The diagram highlights significant differences in the voltage curves across different values of *J*a and R, indicating distinct growth processes of alumina. Characteristic signals for analysis include transition time (*T*), transition point voltage (*U*, *U*′), and the average rate of voltage change (*k*, *k*′) during growth stages I to IV. It is important to note that due to the experimental time constraint of 100 min, some samples did not progress beyond stage III.

**Figure 5:**
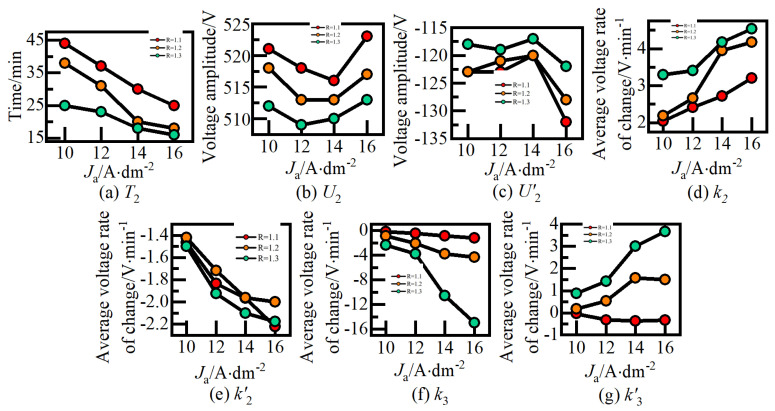
Effect of *J*a on voltage curves at growth stages II, III. (**a**) Transition time of stage III. (**b**) Transition positive voltage of stage III. (**c**) Transition negative voltage of stage III. (**d**) Positive voltage change rate in stage II. (**e**) Negative voltage change rate in Stage II. (**f**) Positive voltage change rate in Stage III. (**g**) Negative voltage change rate in Stage III.

The voltage curves of the 12 sample groups depicted in Figure 4 exhibit minimal divergence in stage I. However, differences in subsequent stages are persistently compared and analyzed. Figure 5 presents a comparative analysis of *T*, *U*, and *k* in the voltage curves of the film-forming process during growth stages II and III under varying *J*_a_ and R values. (a), (b), (d), (f) are positive voltage curve, (c), (e), (g) are negative voltage curve. 

Notably, the change trends of T, U, and k in the negative voltage curve closely mirror those observed in the positive direction. In Figure 5a, it is evident that the *T*_2_ time of the process voltage curve for the ceramic film obtained under *J*_a_ = 10A/dm^2^and *R* = 1.1 occurs at the latest time, 44 min. Conversely, the *T*_2_ time under *J*_a_ = 16A/dm^2^and *R* = 1.3 is the earliest, manifesting at 16 min, preceding the former by 28 min. Notably, the change trends of *T*, *U*, and *k* in the negative voltage curve closely mirror those observed in the positive direction.

When R remains constant, *T*_2_ advances with increasing *J*_a_. Conversely, for a fixed *J*_a_, *T*_2_ advances with increasing *R*. Similarly, *T*′_2_ follows a similar trend. Hence, the onset time for entering the ‘soft spark’ discharge mode can be advanced by appropriately increasing both the current density and the intensity of the negative pulse. In Figure 5c, it is observed that the forward voltage peak *U*_2_ of the ceramic membrane process voltage curve obtained at *J*_a_ = 12 A/dm^2^, *R* = 1.3 is the smallest, measuring 509 V. Conversely, at *J*_a_ = 16 A/dm^2^, *R* = 1.1, *U*_2_ is the largest, reaching 523 V, representing an increase of approximately 2.7%.

Under the same *J*_a_, *U*_2_ decreases with increasing *R*. Conversely, for a fixed *R*, U_2_ follows a *U*-shaped curve in response to the increase in *J*_a_. In the left half of the *U*-shaped curve, characterized by a lower range of *J*_a_, *U*_2_ decreases, primarily influenced by the advance of *T*_2_, resulting in a limited increase in voltage. However, with a further increase in *J*_a_, the growth rate of the ceramic film accelerates, leading to a higher positive electrode breakdown voltage. Figure 5e,g illustrate that the average voltage change rates, *k*_2_ and *k*_3_, of ceramic membranes during growth stages II and III both increase with rising values of *J*_a_ and *R*, ascending from 2V/min and −0.2 V/min to 4.5 V/min and −15 V/min, respectively. Consequently, augmenting *J*_a_ and *R* facilitates rapid growth and deposition of ceramic membranes in stage II, while adjusting the voltage drop in stage III can modulate the growth state of alumina.

**Figure 6:**
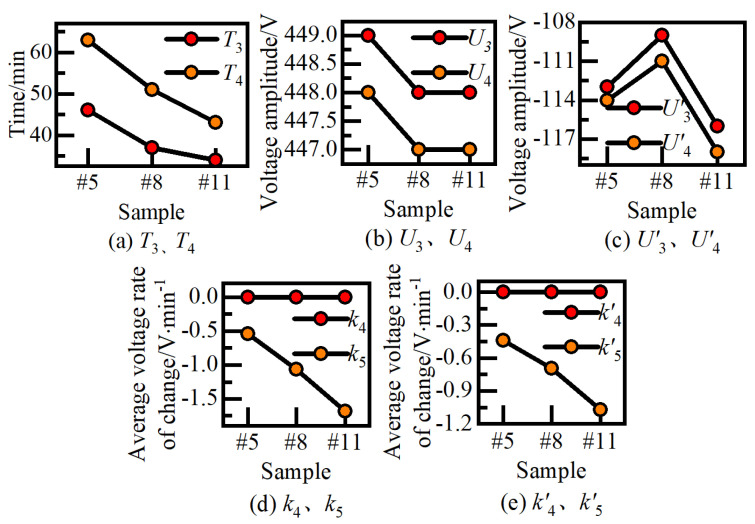
Effect of *J*_a_ on voltage curves at growth stages IV and V. (**a**) Transition time of stage IV and V. (**b**) Transition positive voltage of stage IV and V. (**c**) Transition negative voltage of stage IV and V. (**d**) Positive voltage change rate in Stage IV and V. (**e**) Negative voltage change rate in Stage IV and V.

Figure 4 and Figure 5 examine the effect of positive and negative pulses on the growth process of ceramic films in stages I, II, and III. Next, the influence of different positive and negative pulses on the growth process of ceramic films in stages IV and V was analyzed using samples #5, #8, and #11, where R is 1.2 and *J*_a_ gradually increases. The effect of current density, *J*_a_, on the process voltage in growth stages IV and V (*T*, *U*, *k*) is presented in Figure 6.

In Figure 6, (a), (b), (d) are positive voltage curve, (c), (e) are negative voltage curve. It is notable that the change trend of the negative electrode voltage-time response curve aligns closely with that of the positive electrode process. As depicted in Figure 6, with the increase of *J*_a_, *T*_3_ (*T*′_3_) and *T*_4_ (*T*′_4_) advance at the time of entering stage IV and V, respectively, while the corresponding initial voltage values *U*3 (*U*′_3_) and *U*4 (*U*′_4_) decrease. Furthermore, the average change rates k4 (*k*′_4_) and k5 (*k*′_5_) of stages IV and V increase with the rise of *J*_a_. Notably, *k*_4_ approaches 0, while *k*_5_ varies between −0.5 and −1.7 V/min.

The above findings highlight that the positive and negative current density parameters have a significant impact on the initiation time (*T*), reaction rate, and process voltage value (*U*) of each growth stage. Strengthening the cathode pulse effect or elevating the current density level effectively enhances the reaction conditions of micro-arc oxidation in each stage, leading to the advancement of T and an acceleration in the voltage drop rate (*k*) before entering the soft spark mode. In stages II and III, the initial voltage value (*U*) of each stage initially decreases and then increases with the increase of *J*_a_, while it decreases with the rise of *R*. Conversely, in stages IV and *V*, *U* decreases as *J*_a_ increases. Moreover, a model can be developed based on the corresponding description of the microstructure and properties of the ceramic membrane derived from characteristic voltage curves. This model enables the online evaluation of the film growth state through voltage process monitoring.

### 3.3. Surface Morphology and Structural Molecules of the Ceramic Film under Different Positive and Negative Pulses

**Figure 7:**
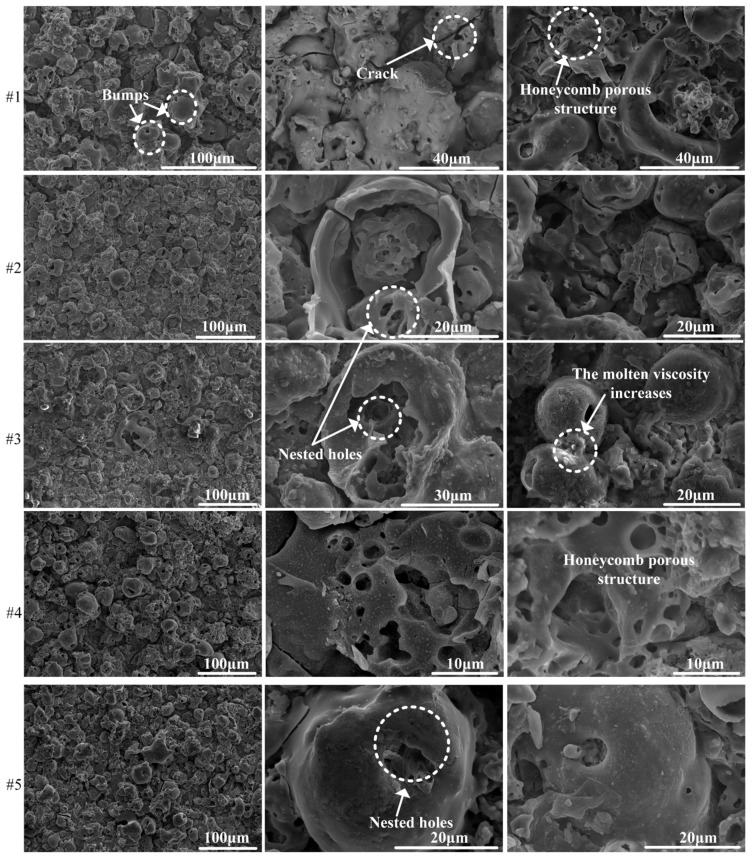

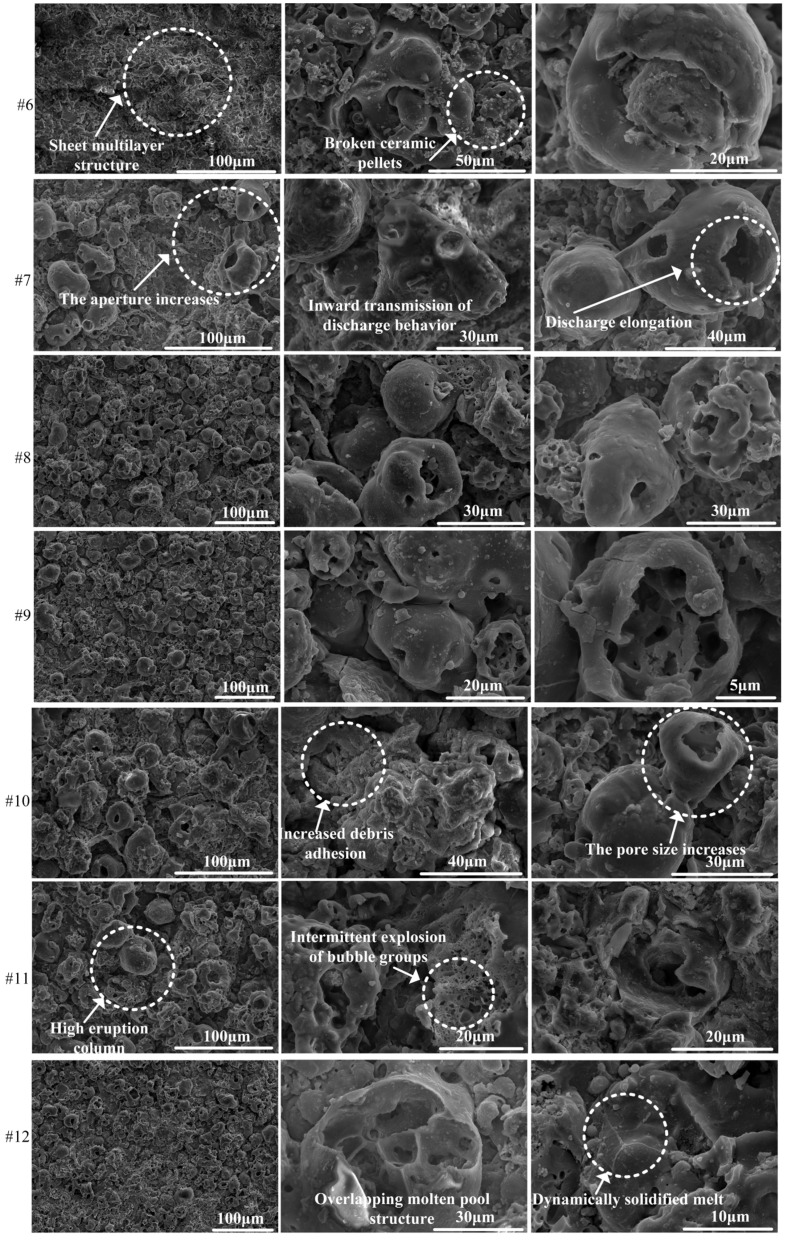
Microscopic morphology of the surface of the micro arc oxidation ceramic film.

Figure 7 presents the surface morphology of 12 groups of ceramic films subjected to different positive and negative pulses. Three images were taken of each sample, increasing in magnification when viewed from left to right. It is evident from the figure that the surface of the ceramic film is rough in all samples and consists of “cake-like” projections, sintered and fused particles, pores and cracks. The bulging micropores serve as channels for electrolyte-matrix reactions and as eruption channels for molten oxides generated during these reactions. The ceramic film, under the high temperature of micro-arc conditions, predominantly centers around these micropores. Oxides continue to melt, rapidly solidify, and amalgamate to form a porous structure. The stress induced by volume shrinkage during melt solidification gives rise to microcracks, with micropores serving as the source of crack initiation [17]. 

When *J*_a_ remains constant, an increase in *R* results in the observation of more layered amorphous regions in the bright regions of the film surface topography. The pore size of the discharge molten pool decreases, while the amount of melt increases. The surface flow distribution morphology becomes more pronounced, with elongated and thin melt flow patterns. The spherical protrusions of the eruption column weaken, with blurred contours, reduced height, and increased debris from attached eruption products.

When *R* remains constant, an increase in *J*_a_ results in a heightening of the eruption column in the volcanic shape. This indicates an augmentation in the gas volume within the discharge channel, leading to a reinforcement of the discharge intensity. Consequently, there is an escalation in the resistance to the outward ejection of the melt during the film-forming process, resulting in increased melting depth, enhanced concavity, and enlarged pore size of the melting pool. Moreover, the discharge behavior propagates through the cavity in a fissure type eruption, further contributing to an increase in the overlapping melt pool structure. This leads to a more abundant spongy porous structure distributed on the surface. Additionally, there is an uptick in the number of ceramic spherical product particles sputtered during the discharge film-forming process.

**Figure 8:**
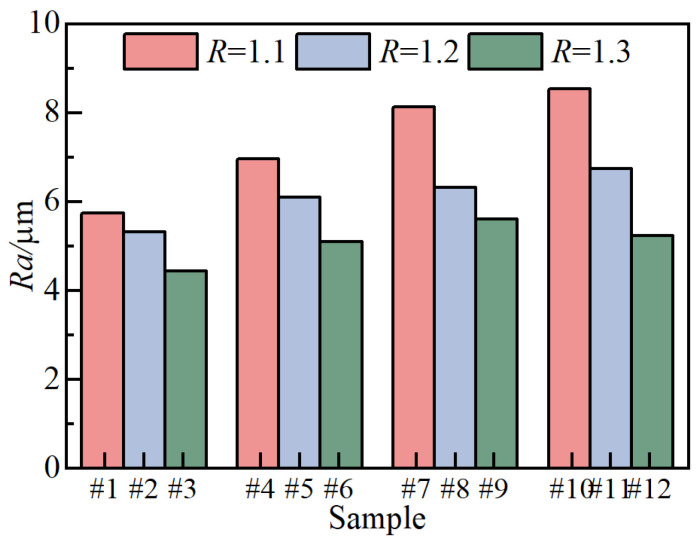
Micro arc oxidation ceramic film surface roughness.

Figure 8 depicts the surface roughness measurement results of 12 groups of ceramic films. The roughness *R*_a_ of the ceramic films falls within the range of 4–8 μm. Notably, sample #10 exhibits the highest roughness, while sample #3 displays the lowest. The roughness value of the ceramic film decreases with increasing R and increases with rising *J*_a_. Upon examination of the surface morphology observed in Figure 8, it can be concluded that the former is attributed to the heightened distribution of molten oxides on the ceramic film surface, while the latter is attributed to the higher number of ceramic ball particles scattered on the surface.

**Figure 9:**
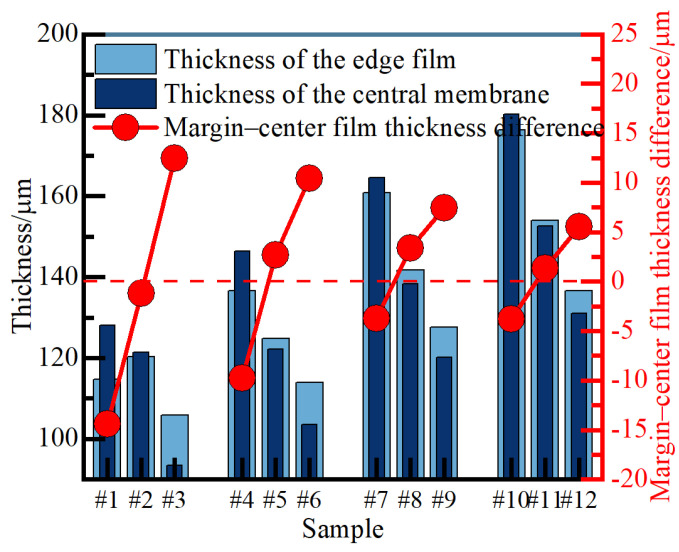
Comparison of film thickness and difference between the center and edge of the ceramic film.

Figure 9 presents a comparison of the center and edge film thickness of the 12 groups of ceramic films, including the difference between the two. The inconsistent film thickness of the ceramic film in different regions stems from the selective growth of the film induced by the uneven distribution of current on the sample surface. The primary factor contributing to the variation in film thickness between the middle plane and the edge is the interface type. While the plane and the electrolyte exhibit two-dimensional unidirectional contact, the edge and the electrolyte involve spatially multi-angle contact. As a result, atoms at the interface deviate from their equilibrium positions to differing extents, leading to an increase in energy. This increase in energy is referred to as interface energy. Notably, the interface energy of the system at the edge surpasses that of the plane area, resulting in an overall increase in the system’s internal energy and a tendency towards stability in the region. On the other hand, the specific surface area of atoms and ions at the edges and vertices exceeds that of the planar region, facilitating the adsorption of negatively charged micelles in the electrolyte and the formation of discharge centers. These factors contribute to the propensity for breakdown and preferential growth at the film edges. As the ceramic film thickness increases, edge growth stops because it becomes more difficult to break down, resulting in a notable growth rate improvement at the center of the film [18]. The results from Figure 9 reveal that the center-edge difference in ceramic film thickness is most pronounced in samples #3 and #1, measuring 15 μm and −15 μm, respectively. Spatial distribution differences in ceramic film thickness are minimal under *R* = 1.2 parameters, nearly approaching 0. When *J*_a_ remains constant, the growth of ceramic films after 100 min of treatment at *R* = 1.1, 1.2, and 1.3 results in intermediate thickness, uniform thickness, and edge thickness, respectively. This may be attributed to the fact that increasing *R* promotes the dissolution reaction (1) of the film layer at the electrolyte/film interface during cathode pulse, thereby slowing the film surface growth rate and inhibiting edge preferential growth mode [19]. From the trend of change, continuing to increase *J*_a_, the thickness difference between the center and the edge of the ceramic film will be infinitely close to zero.

(1) $${Al}_{2}O_{3}+6{H^{+}}_{\left(aq\right)}\to 2{{Al}^{3+}}_{\left(aq\right)}+3{H_{2}O}_{\left(l\right)}$$

By considering the original surface of the aluminum substrate as the reference line, it was observed that the new ceramic coating formed both above and below this line. The region extending from the reference line to the electrolyte/coating interface was designated as the outward growth behavior of the coating, while the region from the reference line to the coating/substrate interface was characterized as the inward growth behavior of the coating. The mechanism for the growth of micro-arc oxidation alumina ceramic coating stems from the movement of Al^3+^ and ions containing oxygen atoms under the influence of a high electric field (O^2−^ and OH^−^). Specifically, Al^3+^ migrates outward through the alumina coating, leading to the formation of a new oxide film at the electrolyte/coating interface. Simultaneously, O^2−^ and OH^−^ ions move inward through the oxide film, resulting in the formation of a new oxide film at the coating/substrate interface [20,21]. The reactions (2), (3), (4) are as follows:

The substrate/film interface:

(2) $$Al\to {Al}^{3+}+3e^{-}$$

(3) $$2{{Al}^{3+}}_{\left(ox\right)}+3{O^{2-}}_{\left(ox\right)}\to {Al}_{2}O_{3}$$

The Film/electrolyte interface in addition to the above reactions, there are

(4) $${{2H}_{2}O}_{\left(l\right)}+2e^{-}\to {2OH}^{-}+{H_{2}}_{\left(aq\right)}$$

**Figure 10:**
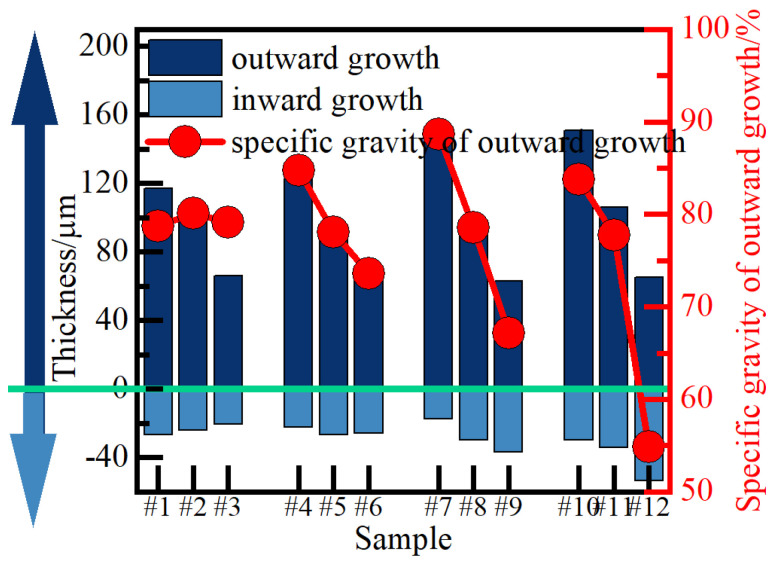
The inward and outward growth of micro-arc oxidation ceramic film.

Figure 10 illustrates the disparity between the internal and external growth of the 12 groups of ceramic membranes. It is evident from the figure that the thickness of the outward growth of the ceramic membrane under different current density parameters exceeds 50% of the total thickness, consistently displaying stronger outward growth behavior compared to inward growth. Of the samples tested, sample #7 had the smallest inward growth thickness and proportion of ceramic membrane, resulting in a matrix erosion ratio of 11%. Conversely, sample #12 exhibits the largest inward growth thickness and proportion, with a matrix erosion ratio of 46%. 

Overall, when *J*_a_ remains constant, an increase in *R* results in an increase in the inward growth thickness of the ceramic film, accompanied by a decrease in outward growth thickness and an increase in substrate erosion ratio. This phenomenon may be attributed to the intensified dissolution reaction at the electrolyte/film interface during the cathode pulse. Consequently, the growth behavior of Al^3+^ outwards and its combination with oxide formation is inhibited, thereby reducing the outward growth rate of the film. As *J*_a_ increases while *R* remains constant, the outward growth thickness and inward growth thickness of the film increase. Specifically, when *J*_a_ is less than or equal to 14 A/dm^2^, increasing *J*_a_, the outward growth thickness of the ceramic film increases more significantly, with the outward growth behavior of the film being the main contributor to the thickness increase. When *J*_a_ reaches 16 A/dm^2^, the thickness of the ceramic film increased even more. 

When *R* remains constant, an increase in *J*_a_ results in a simultaneous increase in both outward and inward growth thicknesses of the film. Particularly, when *J*_a_ ≤ 14 A/dm^2^, elevating *J*_a_ leads to a more significant increase in the outward growth thickness of the ceramic film, with outward growth behavior being the primary contributor to thickness augmentation. However, as *J*_a_ reaches 16 A/dm^2^ and *J*_a_ continues to rise, which will leads to more inward growth thicknesses of the film. This phenomenon can be attributed to several factors. On the one hand, the increase of the output energy during the pulse makes the film/substrate interface produce more molten reactants due to the instantaneous high temperature. On the other hand, the increase of the film thickness may further inhibit the outward migration of Al^3+^.Under the combined action of the two, the ceramic film preferentially grows inward.

**Figure 11:**
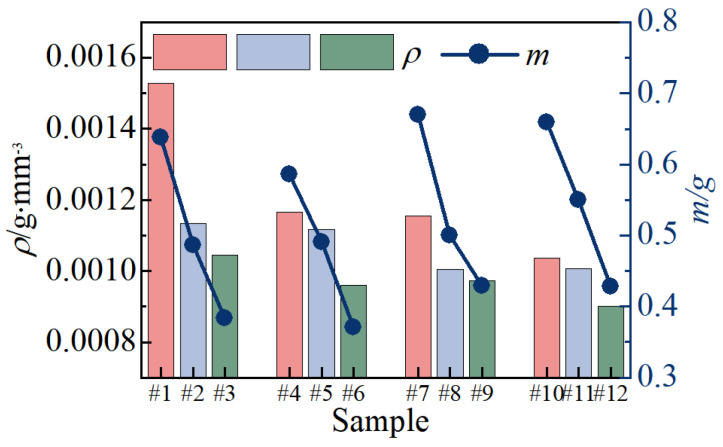
Density and quality of micro-arc oxidation ceramic film.

Figure 11 illustrates the density comparison of 12 groups of ceramic membranes, alongside the corresponding film weight data. The two curves depicting density and film quality exhibit a similar trend with changes in current parameters. Notably, the density of the #1 ceramic film, prepared with *J*_a_ = 10A/dm^2^ and *R* = 1.1, is the highest at 0.0015 g·mm^−3^. Conversely, the density of the No.12 ceramic film, prepared with *J*_a_ = 16 A/dm^2^ and *R* = 1.3, is the lowest, measuring only 0.0009 g·mm^−3^, marking a decrease of 40%. When *J*_a_ remains constant, an increase in R leads to a linear decrease in both the weight and density of the film. This observation suggests that heightened cathode pulse intensity is not conducive to the growth of alumina grains, resulting in decreased film density. Conversely, when *R* is constant, an increase in *J*_a_ enhances the quality of the ceramic membrane but reduces its density. This indicates that while increased pulse energy accelerates the film formation process, the resulting particles are loose, thereby decreasing film density.

**Figure 12:**
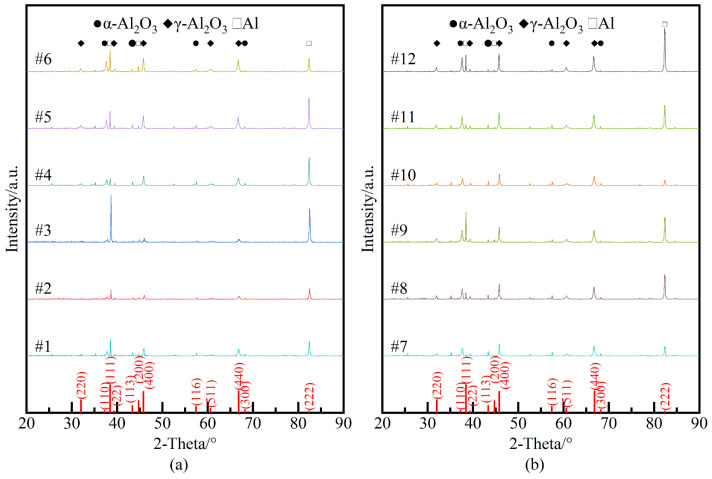
Phase composition of the micro-arc oxidation ceramic film. (**a**) Samples 1 to 6; (**b**) Samples 7 to 12.

Figure 12 depicts the X-ray diffraction pattern of 12 groups of ceramic membranes, revealing that the micro-arc oxidation film primarily comprises α-Al_2_O_3_ and γ-Al_2_O_3_, with γ-Al_2_O_3_ being predominant. The card numbers are 85-1327(Al), 88-0826(α-Al_2_O_3_), 79-1558 and 80-0956(γ-Al_2_O_3_). This dominance of γ-Al_2_O_3_ is attributed to the high temperatures generated during the film formation process, leading to the oxidation of most aluminum alloy surfaces to γ-Al_2_O_3_. Only during the ultra-high temperature stages of intense discharge does a small portion of γ-Al_2_O_3_ convert to α-Al_2_O_3_. 

**Figure 13:**
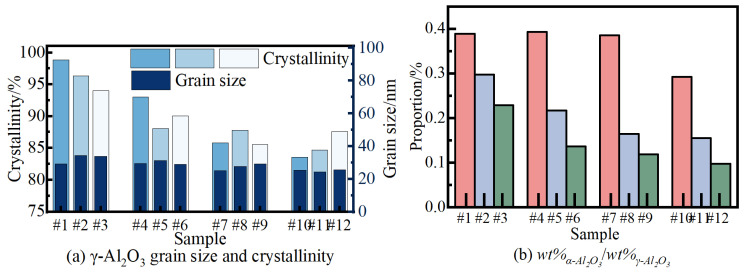
Quantitative analysis of the phase of the micro-arc oxidation ceramic film. (**a**) Crystallinity and grain size of sample#1–#12. (**b**)The proportion of wt%_α-Al2O3_/wt%_γ-Al2O3_ of sample#1–#12.

Based on the results presented in Figure 12, quantitative analysis was conducted using Jade software to determine the crystallinity, grain size, and relative content of the ceramic membrane, as shown in Figure 13. Figure 13a illustrates that the grain size of γ-Al_2_O_3_ crystals in the micro-arc oxidation ceramic film increases with the augmentation of *R* and *J*_a_, ranging from a maximum of 35 nm to a minimum of 25 nm. Conversely, the crystallinity decreases with the increase of R and *J*_a_, reaching a peak of 97% and a low of 84%, respectively. These results suggest that the intensification of cathode pulse action accelerates the dissolution of small grains, facilitating their orientation into larger grains. Furthermore, the augmentation of pulse energy promotes the recrystallization process from small to large grains, thereby increasing the grain size. In Figure 13b, the relative content of α-Al_2_O_3_ and γ-Al_2_O_3_ in the ceramic film decreases from 0.4 to 0.1 with the augmentation of R and *J*_a_. This trend suggests that the conversion of γ-Al_2_O_3_ phase into α-Al_2_O_3_ is reduced, and the increase in *R* and *J*_a_ further diminishes the phase inversion rate. It is important to note that the maximum penetration ability of X-rays is only in the range of tens of microns. Therefore, the discussion of the ceramic membrane’s phase composition pertains to the surface thickness of tens of microns.

### 3.4. The Cross-Sectional Morphology and Structure of the Ceramic Film

**Figure 14:**
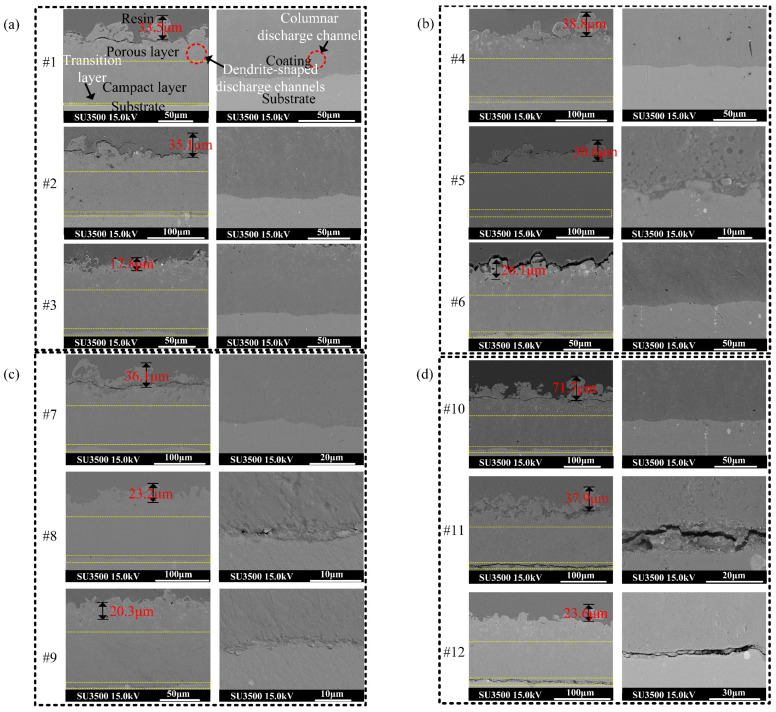
Cross-sectional microscopic morphology from matrix to ceramic film surface. (**a**) Sample#1–#3. (**b**) Sample#4–#6. (**c**) Sample#7–#9. (**d**) Sample#10–#12.

Figure 14 depicts the cross-sectional morphology of the 12 groups of ceramic films along the thickness direction of the film layer. Two images were attached to each sample. The left side presents the overall cross-sectional map from the surface of the film layer to the substrate, while the right side displays a high-magnification map of the interface between the ceramic film layer and the substrate transition layer. As can be seen from the Figure 14a, the cross-section of the film layer can be roughly divided into three regions with different volume percentages: the transition layer, the dense layer, and the surface pore layer. The transition layer, combined with the substrate metallurgy, is only a few micrometers thick. The dense layer which constitutes 60–70% of the total film layer, has a compact structure. The surface pore layer, which has a loose structure, makes up the remaining percentage. Micropores are distributed with varying heights and sizes, resulting in surface unevenness. Discharge reaction channels and residual holes from the reaction are dispersed within the film layer. Furthermore, it is evident that the flatness of the ceramic film surface is primarily influenced by the height of the eruption column of the volcanic morphology of the outer porous layer. 

When the current density (*J*_a_) remains constant, an increase in the pulse voltage (*R*) leads to a tendency towards a flatter volcanic morphology on the surface of the ceramic film. Conversely, when the current density increases while *R* remains unchanged, the fluctuation of the volcanic morphology on the film surface intensifies, consistent with the previously measured changes in roughness. Examining the high-magnification diagram of the interface between the ceramic film layer and the substrate transition layer in the right column of Figure 14 reveals clear wavy boundary structures in the transition layers of most ceramic films. These layers exhibit good bonding states, devoid of structural defects, and are dispersed with irregular blocks and powder oxides. However, the degree of undulation in the wavy boundary varies among ceramic films produced under different current density parameters. For samples #11 and #12, excessive *J*_a_ may lead to concentrated stress from gas release during the discharge growth of ceramic membranes, resulting in the formation of penetrating cracks in the relatively weak surface pore layer and transition layer.

**Figure 15:**
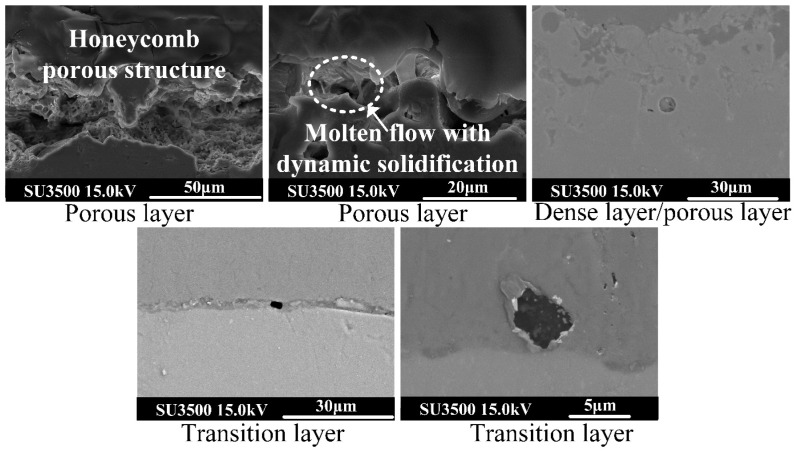
Micropore structure topography of ceramic film cross-section.

Photographs of the cavity structure in each area of the ceramic membrane section were captured using high power electron microscopy, as depicted in Figure 15. The images reveal that the cavity size within the surface porous layer is the largest, exhibiting a honeycomb-like structure, likely resulting from intermittent bubble group explosions. Conversely, the number of cavity structures at the junction of the dense layer and the porous layer, as well as the transition layer near the matrix side, is small, and their size is compact. In Figure 15, a slender body structure formed through dynamic solidification of molten material along the crack is evident, thus corroborating previous observations.

**Figure 16:**
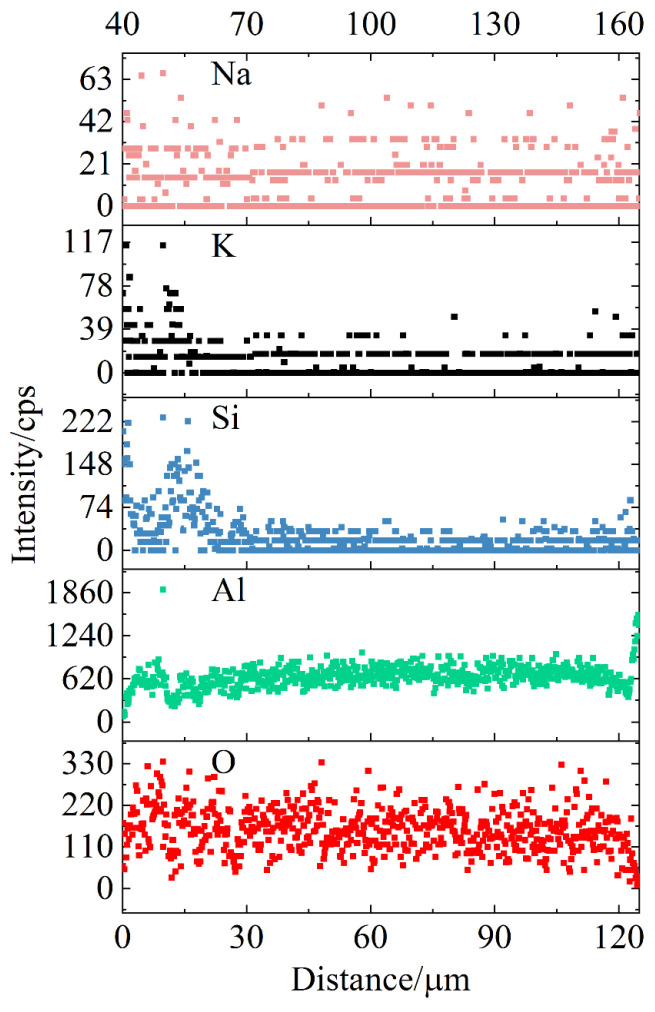
Cross-sectional line scanning EDS analysis of specimen #9.

In accordance with the cross-section SEM image of the #9 ceramic film in Figure 14, a perpendicular line segment was drawn from the film layer’s surface to the substrate side for line-scanning EDS elemental analysis, as shown in Figure 16. The abscissa’s zero point corresponds to the outer surface of the ceramic film near the electrolyte side. Examination of the results reveals that the micro-arc oxidation ceramic film is primarily composed of O and A, with smaller quantities of Si, K and Na from the electrolyte. Notably, the Al element is the most prevalent, constituting over 50%, followed by O. The distribution of O and Na elements is relatively uniform throughout the film, whereas Si and K elements exhibit a distinct peak within the 20 μm wide surface porous layer of the ceramic membrane, with even dispersion in other membrane layer areas. The distribution of Al element increases gradiently in the near-surface area of the ceramic membrane about 2 μm and the transition layer in contact with the matrix. It can also be judged that these two areas are mainly responsible for the subsequent outward and inward growth of the ceramic membrane. There is a valley value of Al element distribution in the film layer corresponding to the peak distribution of Si and K elements. This local unbalanced element distribution may be affected by the formation defects of the porous layer of the ceramic film.

### 3.5. Distribution of Hardness of Cross-Section

**Figure 17:**
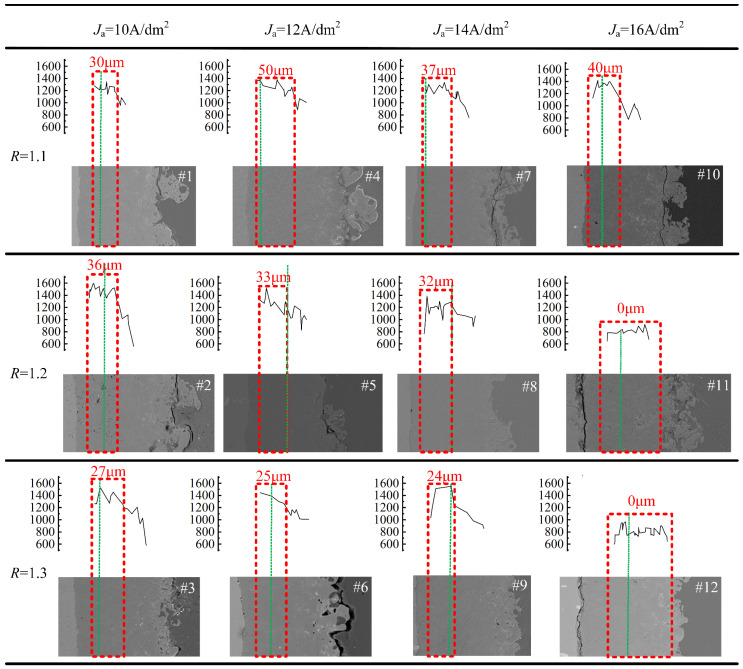
Cross-sectional hardness distribution of ceramic film from matrix to film surface.

Figure 17 illustrates the microhardness distribution across the cross-section of the ceramic film at various current densities, along with the corresponding cross-sectional morphologies. The green lines indicate the original substrate surface before the micro-arc oxidation treatment, while the red wire frames outline areas in the ceramic film cross-section that exceed 1200 HV. Figure 17 shows that the cross-sectional hardness of the 12 groups of ceramic films varies due to the formation of different structures and phase compositions from the inside to the outside of the substrate during the growth of the film. The cross-sectional hardness of the 12 groups of ceramic films in the figure shows the distribution characteristics of soft-hard-soft discontinuous regions. The ceramic film proximate to the outer surface of the original substrate exhibits the highest hardness with minimal gradient change. Apart from the ceramic films from #11 and #12 samples, the other ten groups of films have a hardness exceeding 1400 HV, with a high hardness region extending approximately 20–50 μm near the outer substrate surface (exceeding 1200 HV), constituting approximately 20–30% of the total film thickness. This high-hardness area extends in both directions, with hardness decreasing rapidly. It is suggested that the favorable thermal insulation environment in this region promotes high-quality phase transition from γ-Al_2_O_3_ to α-Al_2_O_3_, significantly increasing α-Al_2_O_3_ content. In accordance with the morphology diagram, it is evident that the high hardness zone extends laterally from both sides of the substrate towards the outer film surface, exhibiting a certain width of columnar crystal morphology. This suggests that the internal temperature uniformity and thermal insulation within this region are superior, while significant temperature gradients exist outside this area. Notably, the columnar crystals formed towards the substrate side are perpendicular to the substrate surface, whereas those formed towards the outer film surface develop in a dendritic morphology. The state of the oxide appears relatively loose, likely attributed to electrolyte penetration into the micropores.

A detailed comparison of the cross-sectional hardness distribution among ceramic films #1 to #10 depicted in Figure 17 reveals distinct patterns. Notably, the ceramic film produced under *J*_a_ = 14 A/dm^2^, *R* = 1.3 exhibits the narrowest high hardness area, measuring 24 μm, while the film derived from *J*_a_ = 12 A/dm^2^, *R* = 1.1 displays the widest high hardness zone, spanning 50 μm—an increase of 108%. This trend suggests that, with a constant *R*, increasing *J*_a_ results in thicker films, limiting the transmission of heat energy from the substrate to the film surface during discharge. Consequently, this phenomenon reduces the α-Al_2_O_3_ phase conversion rate and surface hardness, while concurrently widening the high hardness region within the film. Overall, the average film hardness increases with escalating *J*_a_. 

Conversely, if Ja remains constant, increasing *R* leads to a narrower high hardness zone within the film, accompanied by an increase in depth. This outcome suggests that the outward growth behavior of the ceramic film is inhibited under these conditions. Notably, ceramic films #11 and #12 exhibit the highest hardness of approximately 900 and 800 HV, respectively. This discrepancy may be attributed to the compromised integrity of the transition layer in these samples, resulting in inadequate thermal insulation and hindering the phase transition from γ-Al_2_O_3_ to high hardness α-Al_2_O_3_. Consequently, the overall film hardness is diminished.

## 4. Discussion

The growth process of micro-arc oxidation coating primarily occurs through plasma discharge. Molten material is ejected outward within the discharge channel, which is characterized by high temperature and pressure. Upon cooling, a solidification process of liquid Al_2_O_3_ takes place, accompanied by the transformation of γ-Al_2_O_3_ to α-Al_2_O_3_. The γ-Al_2_O_3_, possessing a spinel structure, is inherently unstable and tends to transition to α-Al_2_O_3_ with a denser lattice arrangement at elevated temperatures (800–1200K). This transition rate increases with higher temperatures. That is

(5) $$\gamma -{\mathrm{Al}}_{2}O_{3}\overset{800\sim 1200K}{\to }\alpha -{\mathrm{Al}}_{2}O_{3}$$

the temperature gradient within the coating drives the transfer of high heat from the molten alumina to-wards the cooler regions of the film. This significant temperature differential results in varying crystal transformation behaviors across the radial direction of the film [22,23].

The experiment conducted in this paper utilizes a rectangular wave pulse power supply signal. This choice is motivated by the favorable characteristics of the rectangular wave, which exhibits well defined rising and falling edges, concentrating the power supply energy and resulting in a very high instantaneous input energy density value. During micro-arc oxidation, the immediate onset of high current density upon current activation leads to a reduction of metal ions at exceptionally high overpotentials, resulting in finer grains within the ceramic film. Simultaneously, the strong breakdown capability of the rectangular wave pulse to the ceramic film enables easy penetration through weak areas of the film layer, facilitating the growth of new film through oxidation reactions. Upon current deactivation, discharge ions near the cathode region return to their initial concentrations, eliminating concentration polarization. This elimination is advantageous for employing high pulse current density in subsequent synchronized pulses, promoting processes such as recrystallization, adsorption, and desorption outside the ceramic film [24].

The heat transfer dynamics during micro-arc oxidation can be accurately described by Fourier’s law, due to the short discharge time and concentrated heat, as shown in Equation (6).

(6) $$dQ=-\lambda dS\frac{\partial t}{\partial n}$$

where dQ is the heat transfer rate, W; dS is the heat transfer area, m^2^; ∂t/∂n is the temperature gradient, °C/m; λ is the thermal conductivity, W/(m·K). It can be seen that the thermal conductivity of the object affects the heat transfer. In addition to the type of object, the thermal conductivity is also related to the microscopic morphology of the object. It is well known that the micro-arc oxidation film is mainly composed of solid alumina and gas filled in the pores. The continuous alumina solid phase is discretely distributed with submicron and micron-scale micropores. The equivalent thermal conductivity can be approximately calculated as follows:

(7) $$k=k_{s}\left(1-v_{g}\right)$$

where $k_{s}$ is the solid phase alumina dielectric where $v_{g}$ is the gas phase medium The presence of micropores in the film reduces its thermal conductivity, establishing a buffer zone for heat transfer between the remelted area and the dense layer. This phenomenon facilitates heat accumulation, leading to the formation of high temperature point heat sources with these micropores as centers. In prolonged high temperature environments, the transformation of γ-Al_2_O_3_ to α-Al_2_O_3_ occurs, resulting in volume shrinkage and the generation of thermal stress within the film. This stress concentration within the dense layer or at the edge of the melt zone ultimately leads to crack formation. These cracks continue to propagate around the stressed positions, with molten material flowing in and transferring heat to the adjacent micro-areas under self-weight action, further promoting phase change. The closer the thermal energy is to the substrate, electrolyte interface, the worse the thermal insulation environment is delivered [25]. From this we know, the multiple thermal cycles cause significant changes in the phase change gradient across the radial direction of the film section, with regions closest to the matrix experiencing the most thermal cycles. This process ultimately leads to the formation of a high hardness area, comprising approximately 20% to 30% of the film width. 

Under the same *J*_a_, the effect of increasing *R* on the phase transition behavior and hardness distribution of ceramic films along thickness can be explained as follows. Initially, the increase of negative current density results in a relatively minor accumulation of negative charge at the film/electrolyte interface during subsequent positive pulses, yielding the comparatively weak electric field. Consequently, the discharge pore diameter decreases, which reduces the cooling rate of the melt on the film surface that interacts with the electrolyte. This, in turn, decreases the likelihood of α-Al_2_O_3_ formation. On the other hand, the cathode pulse intensifies the film dissolution reaction at the electrolyte/film interface, causing a reduction in film thickness and worsening the degradation of the thermal energy storage environment. Consequently, the ratio of γ-Al_2_O_3_ phase to α-Al_2_O_3_ phase on the film surface decreases, resulting in a decline in surface hardness of the ceramic film and the contraction of the high hardness zone in the section hardness distribution. 

Under the same *R*, the effect of increasing *J*_a_ on the phase transition behavior and hardness distribution of ceramic films along thickness can be explained as follows. The elevation of pulse energy enlarges the micropore diameter of the discharge channel, resulting in the formation of a certain number of honeycomb porous structures within the film. Consequently, the film becomes less compact, hindering the accumulation of heat near the surface. This leads to a decrease in the proportion of γ-Al_2_O_3_ phase to α-Al_2_O_3_ on the film surface. Simultaneously, the increase in film thickness effectively enhances heat concentration within the film. Consequently, the surface hardness of the ceramic film decreases, and the high hardness area of the cross-section hardness distribution tends to broaden. Regarding the uniform hardness distribution of #11 and #12 ceramic coatings due to penetrating cracks in the transition layer, the explanation is as follows: The presence of cracks induces a much higher transverse heat flow velocity of the coating parallel to the surface direction compared to the longitudinal velocity perpendicular to the surface direction. This disparity causes the thermal conductivity of the coating to exhibit anisotropy, facilitating rapid equilibrium and homogenization of the coating temperature. Additionally, the coating/substrate interface contributes to cooling during the film forming process, resulting in a rapid temperature drop and insufficient maintenance time for alumina melting. Consequently, the conversion ratio of γ-Al_2_O_3_ to α-Al_2_O_3_ is diminished. As a result, the highest and average hardness values of the ceramic films #11 and #12 decrease from approximately 1400 and 1200 to 900 and 800 HV, respectively.

Increasing the intensity of the negative pulse within the positive and negative pulse sequence can cause the micro-plasma discharge to transition into the soft spark discharge mode. The differences in the microstructure of ceramic films produced under different positive and negative pulses can be explained as follows. Upon the introduction of the negative pulse, localized acidification occurs during the cathodic polarization process, altering the electric double layer structure of the electrolyte near the ceramic film’s surface. This leads to the enrichment of neutral hydrogen complex [H•]ox outside the film [17,26]. Consequently, an increase in conductivity occurs in this region, resulting in a decrease in the anode voltage, with the extent of reduction contingent upon the effective conductivity increase. Subsequent anodic polarization concentrates the electric field within the non-conductive region inside the film. The intensified electric field facilitates ion migration and inward growth behavior of the ceramic film, leading to densification. When *J*_a_ is held constant and R is increased, enhanced cathodic polarization expedites the [H•]ox enrichment process, resulting in more rapid and pronounced voltage drop, a relatively narrower non-conductive region, and a slower film growth rate. Furthermore, the film’s surface is prone to the accumulation of molecular hydrogen, resulting in bubble-like defects and increased roughness.

## 5. Conclusions

(1) Based on the characteristics of the positive voltage during the micro-arc oxidation process, the growth of ceramic films can be divided into five stages. The impact of various positive and negative pulse current densities as well as their ratio, *R*, on the segmented growth process of ceramic films primarily reflected in the transition time, *T*, of each growth stage, the average voltage change rate, k, when entering the soft spark discharge mode, and the dynamic voltage, *U*, during the alumina growth process. 

(2) The microstructure of the micro-arc oxidation (MAO) ceramic film is significantly affected by the pulse current density *J*_a_ and the ratio of positive to negative current den-sity *R*. This variation corresponds directly to differences in the hardness distribution properties of the ceramic film layers.

(3) The ceramic film’s hardness follows a pattern of initially increasing and then decreasing along its thickness direction, with a distinct high hardness region (≥1200 HV) spanning 20–50 μm. Maintaining a constant ratio of positive and negative current density, an increase in current density results in a thicker film, which reduces the degradation of the thermal insulation environment on the film surface. Consequently, both the conversion rate of α-Al_2_O_3_ phase and the surface hardness decrease, while the width of the high hardness zone widens, leading to an overall increase in film hardness. Conversely, maintaining the positive current density constant and increasing the negative current density can restrict outward growth of the film layer, narrowing the high hardness zone and increasing its depth. 

(4) The ceramic films from samples #11 and #12 may have experienced excessive current density (*J*_a_) of 16 A/dm^2^, leading to the generation of concentrated stress during the discharge growth process. Gas release may have worsened this stress, causing the creation of deep cracks in the weaker surface pore layer and transition layer. This has compromised thermal insulation and severely impeded to the phase transition from γ-Al_2_O_3_ to α-Al_2_O_3_ phase with the higher hardness. Consequently, both the maximum and average hardness decrease from approximately 1400 and 1200 to 900 and 800 HV, respectively.

## Figures and Tables

**Table 1:** Current density of positive and negative pulse.

Sample	$J_{a}/A\cdot$dm^−2^	$J_{c}/A\cdot$dm^−2^	*R*
#1	10	11.0	1.1
#2	10	12.0	1.2
#3	10	13.0	1.3
#4	12	13.2	1.1
#5	12	14.4	1.2
#6	12	15.6	1.3
#7	14	15.4	1.1
#8	14	16.8	1.2
#9	14	18.2	1.3
#10	16	17.6	1.1
#11	16	19.2	1.2
#12	16	20.8	1.3

## Data Availability

The data presented in this study are not available due to privacy.
